# Early CT features of COVID-19 pneumonia, association with patients’ age and duration of presenting complaint

**DOI:** 10.1186/s43055-021-00539-5

**Published:** 2021-07-06

**Authors:** Reem M. EL Kady, Hosam A. Hassan, Tareef S. Daqqaq, Rania Makboul, Hanan Mosleh Ibrahim

**Affiliations:** 1 Department of Radiology, Assiut University, Assiut, Egypt; 2 Department of Radiology, Taibah University, Medina, Saudi Arabia; 3 Department of Pathology, Assiut University, Assiut, Egypt; 4 Department of Public Health and Community Medicine, Cairo University, Cairo, Egypt; 5 Department of Family and Community Medicine, Taibah University, Medina, Saudi Arabia

**Keywords:** COVID-19, Typical CT imaging features, Atypical CT imaging features

## Abstract

**Background:**

Coronavirus disease (COVID-19) is a respiratory syndrome with a variable degree of severity. Imaging is a vital component of disease monitoring and follow-up in coronavirus pulmonary syndromes. The study of temporal changes of CT findings of COVID-19 pneumonia can help in better understanding of disease pathogenesis and prediction of disease prognosis. In this study, we aim to determine the typical and atypical CT imaging features of COVID-19 and discuss the association of typical CT imaging features with the duration of the presenting complaint and patients’ age.

**Results:**

The lesions showed unilateral distribution in 20% of cases and bilateral distribution in 80% of cases. The lesions involved the lower lung lobes in 30% of cases and showed diffuse involvement in 58.2% of cases. The lesions showed peripheral distribution in 74.5% of cases. The most common pattern was multifocal ground glass opacity found in 72.7% of cases. Atypical features like cavitation and pleural effusion can occur early in the disease course. There was significant association between increased number of the lesions, bilaterality, diffuse pattern of lung involvement and older age group (≥ 50 years old) and increased duration of presenting complaint (≥ 4 days). There was significant association between crazy-paving pattern and increased duration of presenting complaint. No significant association could be detected between any CT pattern and increased patient age.

**Conclusion:**

The most common CT feature of COVID-19 was multifocal ground glass opacity. Atypical features like cavitation and pleural effusion can occur early in the course of the disease. Our cases showed more extensive lesions with bilateral and diffuse patterns of distribution in the older age group and with increased duration of presenting complaint. There was a significant association between crazy-paving pattern and increased duration of presenting complaint. No significant association could be detected between any CT pattern and increased patient age.

## Background

On December 31, 2019, many cases with pneumonia of unidentified cause have been reported in Wuhan, China [1]. On January 7, 2020, coronavirus disease 2019 (COVID-19) was confirmed as the cause of these reported cases [2]. With rapid worldwide viral transmission, a global pandemic was announced by WHO on January 30, 2020 [3]. Coronavirus disease (COVID-19) is a respiratory syndrome with a variable degree of severity, ranging from a mild upper respiratory tract illness to severe pneumonia and acute respiratory distress [4]. The analysis of genetic sequence discloses that COVID-19 belongs to the β-coronavirus genus, with a 79.0% nucleotide identity to severe acute respiratory syndrome coronavirus (SARS-CoV) and 51.8% identity to Middle East respiratory syndrome coronavirus (MERS-CoV) [5]. The standard of reference for confirming COVID-19 infection depends on microbiological tests such as real-time reverse-transcriptase polymerase chain reaction (rt RT-PCR). However, these tests may not be available in an emergency setting or convey a high rate of false-negative results. The addition of an imaging tool as computed tomography (CT) to the patient diagnostic workup could be helpful in certain clinical scenario [6]. Besides, imaging is a vital component of disease progression monitoring and follow-up in coronavirus-related pulmonary syndromes [7]. The study of temporal changes in the CT findings of COVID-19 pneumonia and the effect of patient age on the imaging features can help in understanding the disease pathogenesis and prediction of disease prognosis. Recent studies have discussed the common CT imaging features of COVID-19 pneumonia; however, only few studies discussed the association of different CT imaging features with patients’ age and the duration of present complaint. In this study, we aim to determine the typical and atypical CT imaging features of COVID-19 pneumonia and discuss the association of common CT imaging features with patients’ age and the duration of the presenting complaint.

## Methods

This is an institutional review board approved study. The informed consent was waived. A retrospective review of the hospital information system was done for all confirmed cases of COVID-19 with available chest CT done early in the course of the disease (within 10 days duration from the beginning of the symptoms). In our center, all suspected cases underwent plain X-ray chest examination, and then only cases with positive or equivocal findings in the chest radiograph were directed to perform CT chest examination. All cases were confirmed by respiratory samples tested by real-time reverse-transcriptase-polymerase chain reaction rt (RT-PCR). All cases with chronic cardiac or thoracic illness were excluded. Available clinical data including patient age, patient’s presenting complaint, duration of the complaint, and clinical index score (mild, moderate, severe) were recorded. The patients were classified according to age into two groups (< 50 and ≥ 50 years old). The patients were classified according to the duration of complaint into ≤ 4 days and > 4 days duration. Data collection was done for a three-consecutive-month duration with a final cohort of 110 cases was identified for our study.

### CT protocol

All included cases underwent CT examination using Philips CT scanner (ingenuity core 64 specification). The examination was done in a supine position with the head advanced. The examination was done during the end of inspiration after breath-hold. Contrast media was administrated only in one case. The used parameters were as follow: Number of detectors 32, tube voltage 120 kV, mAs 300, beam collimation width 64 × 0.625 pitch 0.789, gantry tilt 0, field of view 350. Axial and multiplanar reconstruction images were done.

### Image analysis

Imaging findings were reviewed by 2 radiologists with more than 15 years’ experience. Both readers interpret CT images independently and blindly to clinical information. The CT lesions were evaluated for unilateral or bilateral distribution, lobar involvement (upper, middle, lower, or diffuse) and central or peripheral involvement. CT findings were classified into typical or common patterns (multi focal ground-glass opacity (GGO), GGO with superimposed consolidation, consolidation predominant, linear opacities, peribronchial thickening, and crazy paving), atypical or less common patterns (single GGO, air bronchogram, pulmonary nodule, cavity formation, tree in bud sign, fibrotic changes), and other findings, e.g., vascular enlargement, vascular thrombosis, pleural thickening, pleural effusion, and lymphadenopathy.

Imaging findings were recorded and correlated with available clinical data.

### Statistical analysis

Data were analyzed using SPSS version 21. Inter-rater agreement was calculated using Cohen’s kappa coefficient (j) which is calculated as follows: j = (p0 pe)/(1 pe), where p0 is the observed proportion of agreement and pe is the expected proportion of agreement. Descriptive statistics were displayed using frequencies and percentages for categorical variables and mean ± standard deviation for continuous variables. Chi-square test was performed to investigate the association of patients’ radiological findings with the patients’ age (less than 50 years and 50 years or more) and duration of presenting complaint (4 days or less, more than 4 days). Statistical significance was set at a P value < 0.05.

## Results

A total number of 110 patients, 87 males (79.1%) and 23 females (20.9%) with confirmed COVID-19 infection were retrospectively enrolled in our study. All patients were adults with an age range from 18 to 83 years old (with mean age = 43.5 years old). Seventy-three patients (66.4%) were less than 50 years old and 37 patients (33.6%) were 50 years old or older. The main complaints of most of our patients were fever, cough, sore throat, and body aches. Twenty-five (22.7%) patients had dyspnea or shortness of breath. The duration of complaint at the time of presentation ranged from 2 to 7 days (with a mean duration of 3.7 days). The duration of presenting complaint was 4 days or less in 86 patients (78.2%) and more than 4 days in 24 patients (21.8 %). The clinical index was low in 2 (1.8%) patients, moderate in 35 (31.8%) patients, and high in 73 (66.4%) patients.

As regard the interrater reliability in this study, there was an almost perfect agreement in the assessment of CT images between the two readers (0.93). CT showed abnormal findings in all 110 cases. As regards the distribution of the lesions, the lesions showed unilateral distribution in 22 (20%) cases and bilateral distribution in 88 (80%) cases. For lobar involvement, the lesions were mainly involving upper lung lobes in 10 (9.1%) cases, middle lung lobe and lingula in 3 (2.7%) cases, lower lung lobes in 33 (30%) cases, and showed diffuse involvement in 64 (58.2%) cases. The lesions showed perihilar distribution in only one case (0.9%), peripheral distribution in 82 (74.5%) cases, and diffuse central and peripheral in 27 (24.5%) cases (Table 1).

**Table 1:** Lesions distribution in the study cohort

Distribution	Frequency	Percentage
**Unilateral/bilateral**		
Unilateral	22	20%
Bilateral	88	80%
**Craniocaudal/lobar**		
Upper	10	9.1%
Middle	3	2.7%
Lower	33	30%
Diffuse	64	58.2%
**Transverse**		
Central/perihilar	1	0.9%
Peripheral	82	74.5%
Diffuse	27	24.5%

**Fig. 1:**
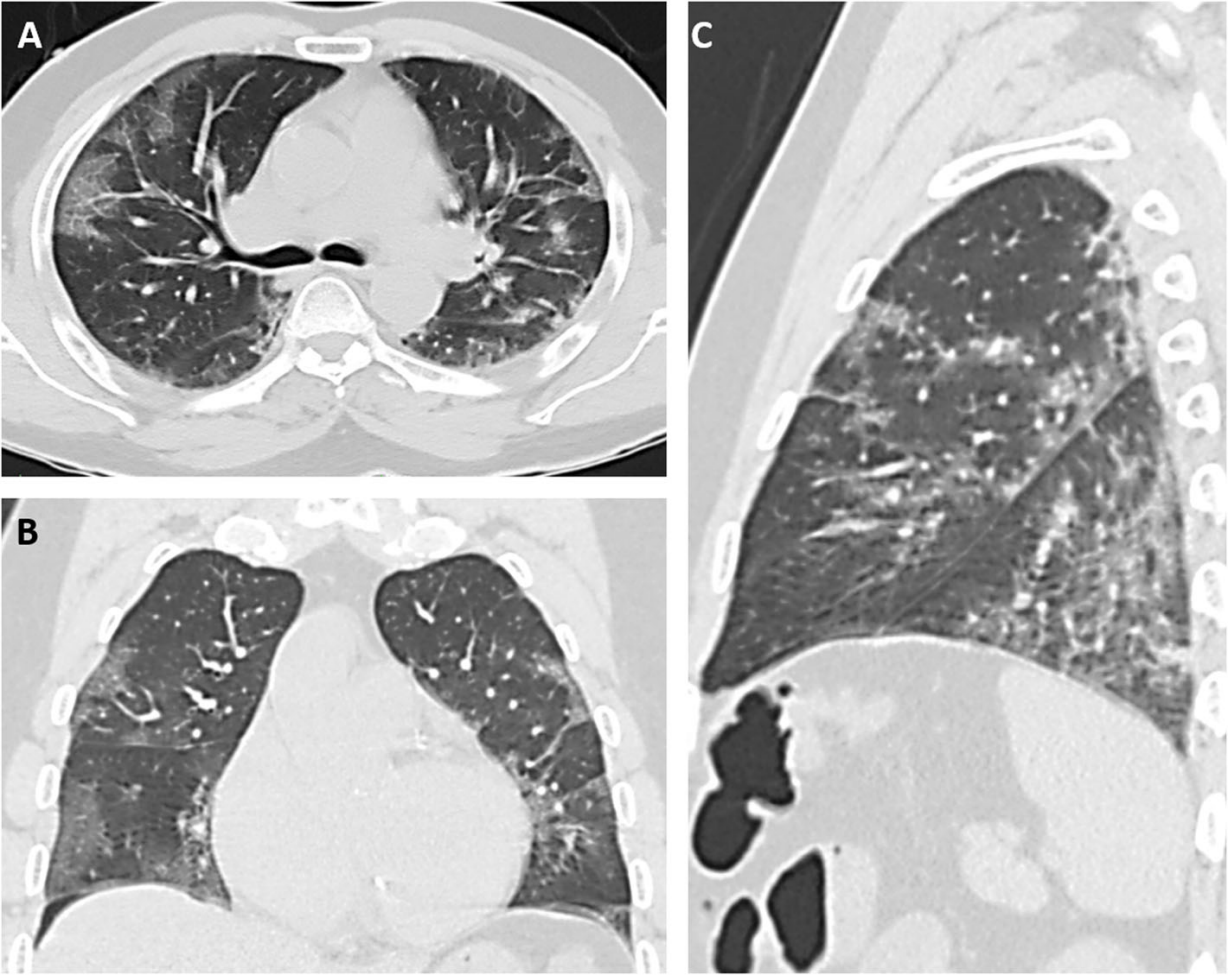
Non-contrast CT of a 42-year-old male with fever, sore throat, and shortness of breath, rt RT-PCR +VE, (**A**-**C**): **A**, axial; **B**, coronal; and **C**, sagittal non-contrast CT scan lung window show bilateral multifocal peripheral and pleural-based ground glass opacity with linear opacities and interlobular thickening

**Fig. 2:**
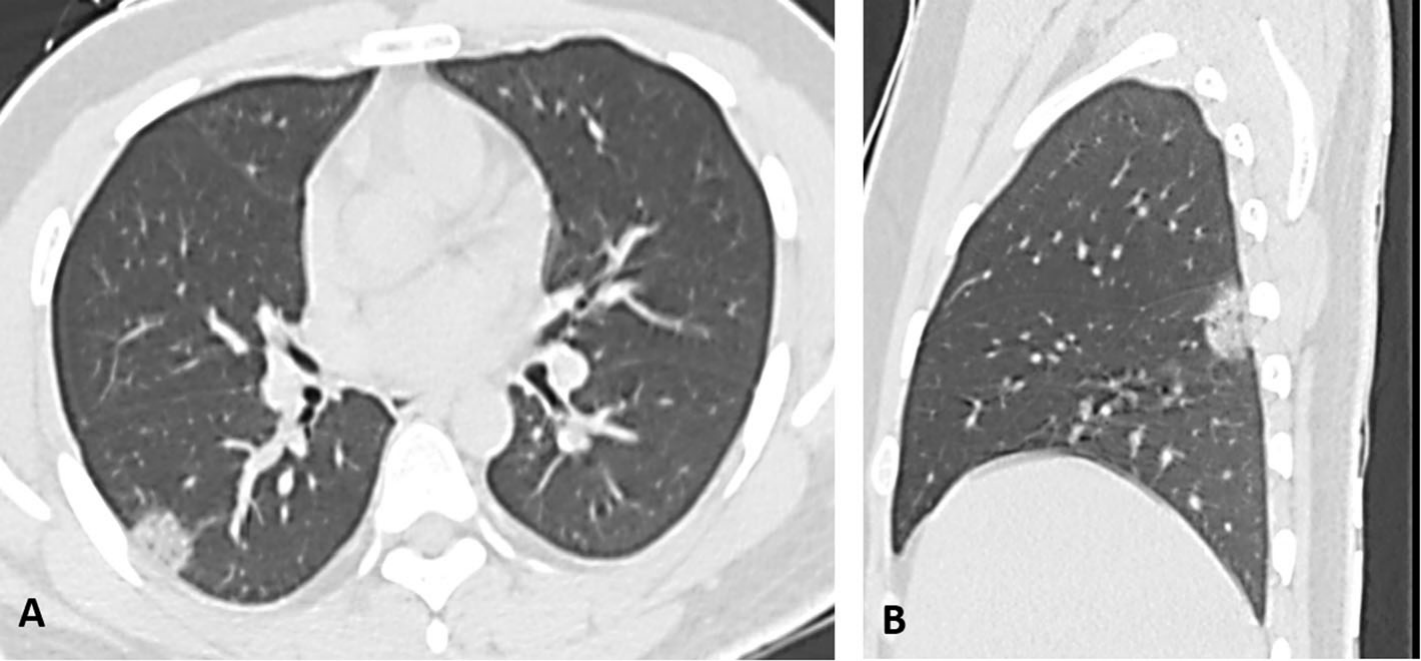
Non-contrast CT of a 36-year-old male with fever, sore throat, and body ache, rt RT-PCR +VE (**A** and **B**): **A**, axial and **B**, sagittal non-contrast CT scan in lung window show peripheral single ground glass opacity in the apical segment of the right lower lobe

**Fig. 3:**
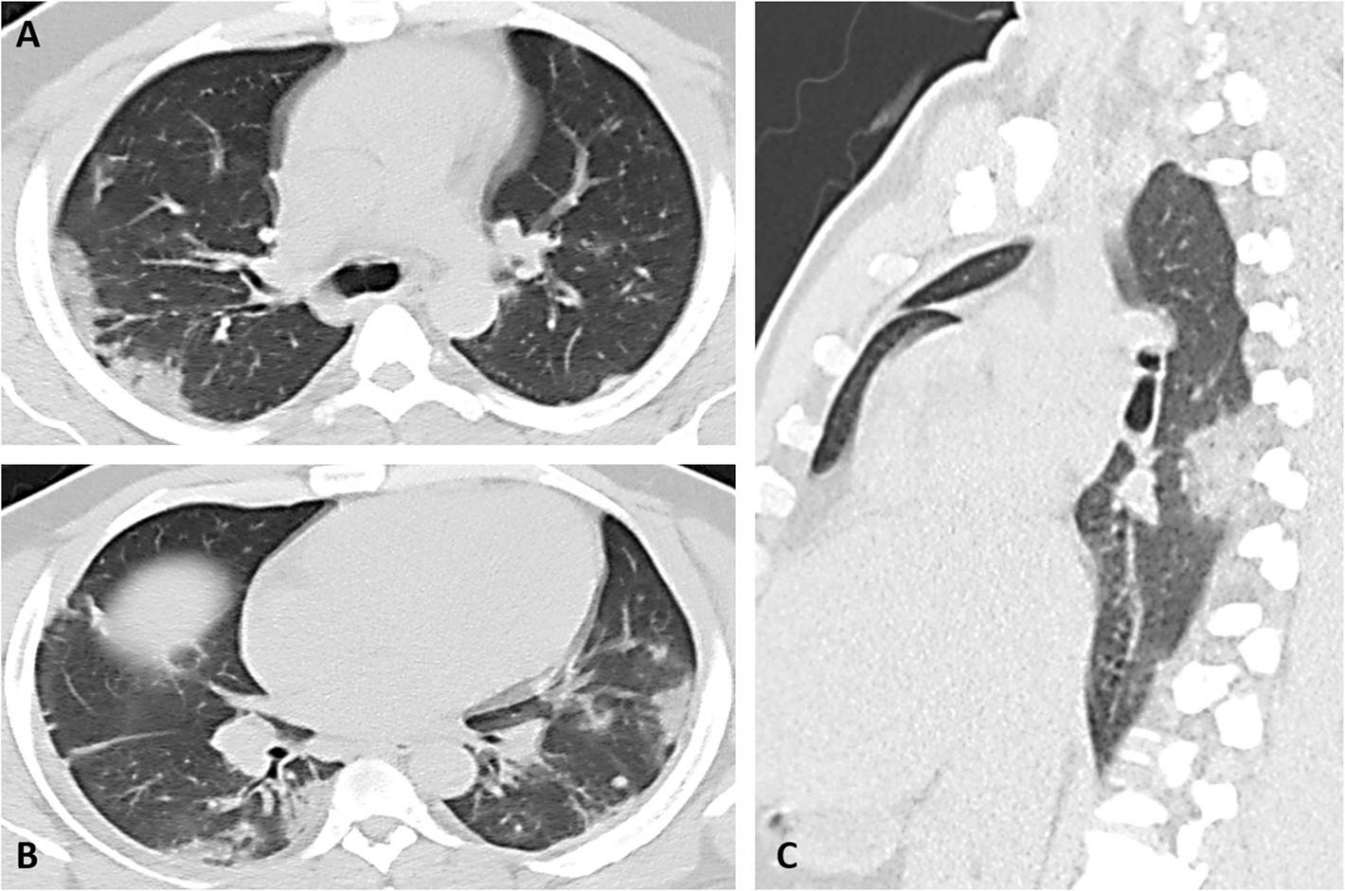
Non-contrast CT of a 52-year-old male with fever and shortness of breath, rt RT-PCR +VE (**A**-**C**): **A** and **B** axial, and **C** sagittal non-contrast CT scan lung window show bilateral multiple subpleural areas of consolidation associated with linear opacities and pleural thickening

**Fig. 4:**
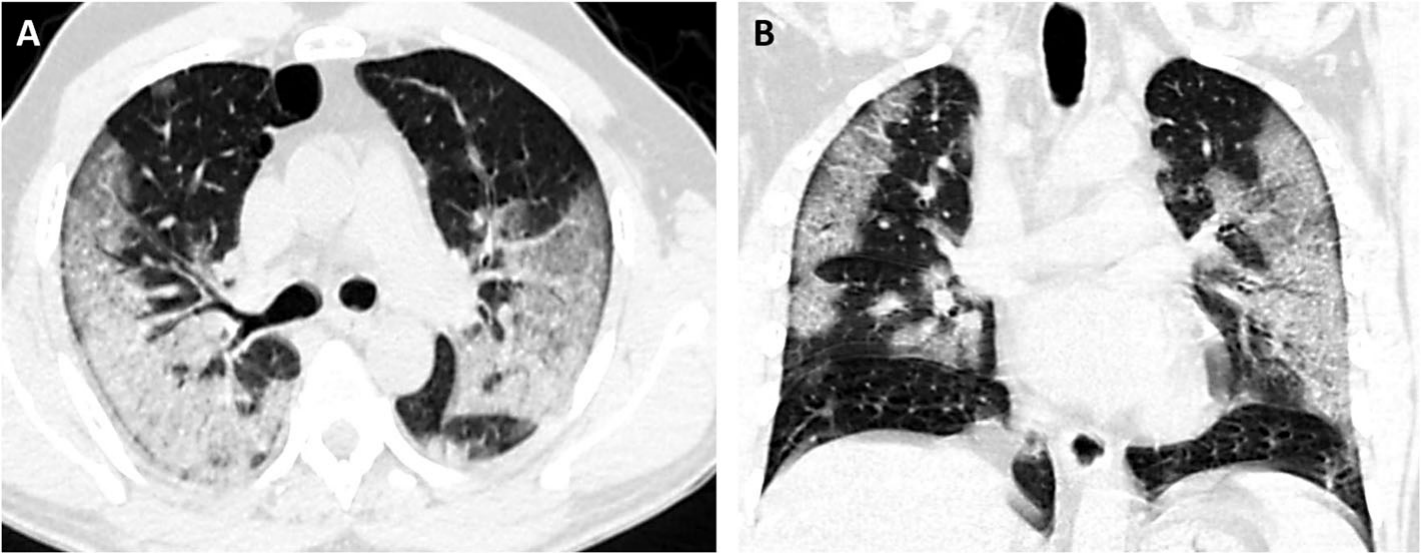
Non-contrast CT done 6 days after initial complaint of a 68-year-old male with high grade fever and tachypnea, rt RT-PCR +VE. (**A**, **B**): **A**, axial and **B**, coronal non-contrast CT scan lung window show bilateral peripheral ground glass density with thickened interlobular and intralobular lines (crazy-paving pattern) involving upper, middle lobes, and lingula

**Fig. 5:**
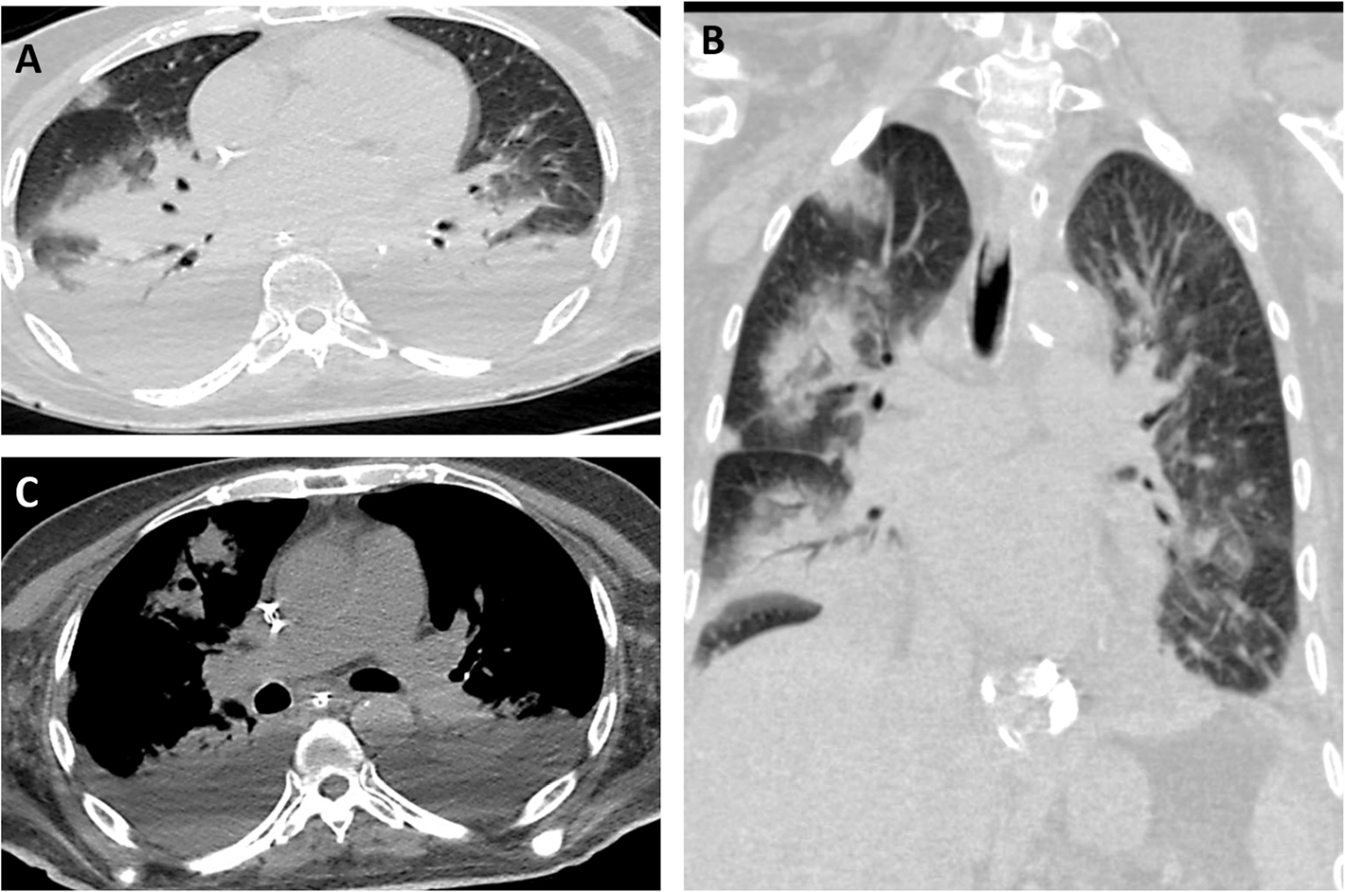
Non-contrast CT done 5 days after initial complaint of a 60-year-old male with fever, cough, body aches, and shortness of breath. rt RT-PCR +ve (**A**-**C**): **A**, axial and **B**, coronal lung window and **C**, axial mediastinal window show bilateral perivascular consolidation (organizing pneumonia pattern) with air bronchogram sign involving both middle and lower lobes with bilateral pleural effusion

The most common pattern was multifocal ground-glass opacity found in 80 (72.7%) cases (Fig. 1), while single ground-glass opacity was found only in 2 (1.8%) cases (Fig. 2). Mixed GGO with consolidation was identified in 27 (24.5%) cases and predominant consolidation in 8 (7.3%) cases (Fig. 3). Pulmonary nodules are detected in only 2 (1.8%) cases and cavitary lesions in 3 (2.7%) cases. Linear opacities were identified in 45 (40.9%) cases, peribronchial thickening in 20 (18.2%) cases, and crazy paving in 10 (9.1%) cases (Fig. 4), air bronchogram sign in 5 (4.5%) cases, and tree in bud sign in 1 (0.9) case. Focal pleural thickening and pleural effusion were identified in 2 (1.8%) and 5 (4.5%) cases, respectively (Fig. 5). Vascular enlargement was identified in 18 (16.4%) cases with only one case (0.9%) showed thrombosis of the right main pulmonary artery. No hilar or mediastinal adenopathy was identified in our cases.

All identified CT patterns are shown in Table 2.

**Table 2:** Different CT imaging findings

	Frequency	Percentage
**Common patterns**		
Multifocal ground glass	80	72.7%
Mixed GGO with consolidation	27	24.5%
Consolidation predominant pattern	8	7.3%
Linear opacities	45	40.9%
Peribronchial wall thickening	20	18.2%
Crazy-paving pattern	10	9.1%
**Less common patterns**		
Single ground glass opacity	2	1.8%
Pulmonary nodules	2	1.8%
Cavitary lesions	3	2.7%
Air bronchogram sign	5	4.5%
Tree in bud sign	1	0.9%
Fibrotic changes	0	0%
**Other findings**		
Focal pleural thickening	2	1.8%
Pleural effusion	5	4.5%
Vascular enlargement	18	16.4%
Vascular thrombosis	1	0.9%
Hilar or mediastinal adenopathy	0	0

As regards association of common CT pattern of COVID-19 with duration of presenting complaint, there was positive association between number of the lesions and bilateral involvement of the lung with the duration of presenting complaint. The peripheral distribution of the lesions was associated with the short duration of presenting complaint (< 4 days) while diffuse pattern of distribution was associated with a relatively longer duration of presenting complaint (≥ 4 days). As regard the association of typical imaging lesions with the duration of presenting complaint, the crazy-paving pattern was detected in (6/24) 25% of patients with duration of presenting complaint > 4 days while was detected in (4/86) 4.7% of patients with duration of presenting complaint ≤ 4 days implying a significant association between crazy-paving pattern and increased duration of presenting complaint (p value = 0.002). No significant association could be detected between the other typical CT features of COVID-19 pneumonia and the duration of presenting complaint.

As regards association of common CT pattern of COVID-19 with patients’ age, there was an association of bilaterality of the lesions and diffuse lung involvement with the older age group (≥ 50 years old). Our cases showed more extensive lesions in older age group (≥ 50 years old) than younger age group (< 50 years old). No significant association could be detected of any typical CT pattern with increased patient age. The associations between common CT pattern detected in our study with duration of presenting complaint and patients’ age are shown in Tables 3 and 4 respectively.

**Table 3:** The association between common CT pattern with duration of presenting complaint

Common CT pattern	Duration ≤4 days (n)		Duration > 4 days (n)		P value
	No	Perc.#	No	Perc.#	
Multifocal ground glass	61	70.9%	19	79.2%	0.423
Mixed GGO with consolidation	21	24.4%	6	25.0%	0.953
Consolidation predominant pattern	8	9.3%	0	0.0%	0.121
Linear opacities	34	39.5%	11	45.8%	0.579
Bronchial wall thickening	17	19.8%	3	12.5%	0.414
Crazy-paving pattern	4	4.7%	6	25.0%	0.002

*Perc.#* percentage within duration group

Statistical significance was set at a P value < 0.05

**Table 4:** The association between common CT pattern with patients’ age

Common CT pattern	Age < 50 (n = 73)		Age ≥ 50 (n = 37)		P value
	No	Perc.*	No	Perc.*	
Multifocal ground glass	48	65.8%	32	86.5%	0.24
Mixed GGO with consolidation	19	26.0%	8	21.6%	0.648
Consolidation predominant pattern	5	6.8%	3	8.1%	0.810
Linear opacities	28	38.4%	17	45.9%	0.539
Bronchial wall thickening	14	23.3%	6	8.1%	0.076
Crazy-paving pattern	4	5.5%	6	16.2%	0.083

Perc.* percentage within age group

Statistical significance was set at a P value < 0.05

## Discussion

CT imaging has a vital role in the diagnosis and management of patients with COVID-19 infection. It allows objective evaluation of the lung lesions, which provides a better understanding of the disease pathogenesis [8].

In general, CT findings of viral pneumonia are diverse and usually affected by the immune status of the host and the underlying pathophysiology of the viral pathogen [9]. Upon entering the pulmonary cells, coronaviruses cause cell damage and pathological changes through direct cytotoxic effects and immunopathogenic effects. These changes are characterized by diffuse alveolar damage (DAD), interstitial mononuclear inflammatory infiltrates, hyaline membrane disease, and desquamation consistent with acute respiratory distress syndrome (ARDS) [10].

Most types of viral pneumonia show bilateral distribution with multiple lung lobes involvement [7]. The lesions in our study showed bilateral distribution in 80% of cases, and unilateral distribution in 20% of cases. For craniocaudal involvement, the lesions were mainly involving the upper lung lobes in 9.1% of cases, the middle lung lobe, and lingula in 2.7% of cases, the lower lung lobes in 30% cases, and showed diffuse involvement in 58.2% of cases. The lesions showed perihilar distribution in only one case (0.9%), peripheral distribution in 74.5% of cases, and diffuse central and peripheral in 24.5% cases. Our results as regards lesion distribution are in line with the previous studies [6, 7, 11–19]. The bilateral peripheral basal distribution which is often seen in many viral pulmonary diseases could be explained by the small size of pathogenic microorganism particles and its brisk way to reach peripheral tissue and attack the alveolar epithelium.

Based on previous reports, the most frequently observed CT features with coronaviruses affection are diffuse airspace opacities which present as GGO, consolidation, or mixed GGO and consolidation [9, 20–23]. The pathological basis of the airspace opacities in viral pneumonia is diffuse alveolar damages including intra-alveolar edema, fibrin, and variable cellular infiltrates with a hyaline membrane that is usually present early in the course of coronaviruses affection [9, 21]. In a study done by Henckel et al. for 14 confirmed cases of COVID-19 with antemortem CT and autopsy correlation, the histopathological observations of airspace opacities were consistent with diffuse alveolar damage associated with capillary dilatation and congestion [24]. They attributed consolidation and bronchial wall thickening to superimposed acute bronchopneumonia.

In our study on confirmed cases of COVID-19, the most common pattern was multifocal ground glass opacity found in 72.7% of our cases while single ground glass opacity was found only in 1.8%. Mixed GGO with consolidation was identified in 24.5% of the cases and predominant consolidation in 7.3%. Associated bronchial wall thickening was observed in 18.2% of cases. The lesions’ shape varied from round to irregular or confluent patches.

Our findings as regards typical CT features of COVID-19 are matching the other recently published reports about COVID-19 imaging [6, 15, 17–19, 24–26].

With the progression of the disease, the congestion of alveolar septal capillaries and exudation of the fluid into the interstitium cause interstitial septal thickening with linear opacities. Thickened interlobular and intralobular lines in combination with a ground glass pattern are called a crazy-paving pattern. In our study, we included CT exams done within 10 days from the first complaint. Linear opacities and thickening of interstitial septa were observed in 40% of our cases and crazy-paving pattern was observed in 9.1% of cases.

Atypical CT features of COVID-19 are not discussed in depth in most of the previous studies. The atypical imaging features of COVID-19 reported by previous studies include tree in bud and centrilobular nodule, cavitation, vascular thrombosis, predominant perihilar ground-glass opacity, pleural thickening and effusion, pneumothorax, and mediastinal or hilar adenopathy [27–29].

The incidence of pulmonary nodules in COVID-19 as reported by previous studies is ranging from 3 to 13% [28]. In our study, we had two cases that showed pulmonary nodules (1.8%). The nodules were associated with other typical findings in both cases. Lung cavitation is an uncommon finding in COVID-19 pneumonia and usually seen in the late stage [30]. Based on autopsy reports, the cavitation in COVID-19 pneumonia is usually caused by diffuse alveolar damage, intra-alveolar hemorrhage, and parenchymal necrosis [31, 32]. We had 3 cases of lung cavitation in our study (2.7%) with all detected within short duration less than 4 days.

Pleural effusion is relatively rare in cases of viral infection. A study by Shi et al. indicated that the prevalence of pleural effusion varies depending on the stage of the disease. They reported a prevalence of 13% in the third week of illness [15]. Another study suggested that the presence of pleural effusion is a poor prognostic indicator for COVID-19 patients [33]. We had 5 cases with pleural effusion in our study (4.5%) and two cases of pleural thickening (1.8%). The amount of pleural effusion was mild to moderate, 4 of them showed associated predominant consolidation, and one was associated with a mixed pattern of GGO and consolidation. Since we excluded cases with other comorbidities from our cohort, pleural effusion in our cases can be attributed to COVID-19 infection or superimposed bacterial infection. All the cases with pleural effusion in our study presented within 4 days after the presenting complaint and 3 of them were older than 50 years old.

Intravascular microthrombi were noticed in patients with COVID-19, and the combination of DAD and thrombosis is associated with rapid deterioration of clinical conditions in severe COVID-19 cases [34]. In our study, vascular dilatation was detected in 18 cases and pulmonary embolism was detected in one case. The increased incidence of pulmonary embolism in cases of COVID-19 could be attributed to the cytokine storm which causes a release of proinflammatory cytokines that predispose to coagulopathy [35].

The study of temporal changes in the CT findings of COVID-19 pneumonia can help in understanding the disease pathogenesis and prediction of disease prognosis. A study done by Pan et al. revealed that chest CT showed the most extensive disease almost 10 days after symptom onset [14]. Previous studies indicated that the lesions were limited to single or multiple areas and were distributed along the sub-pleural areas in the early phase of the disease. With disease progression, the lesions increased in number and extended gradually from the periphery to the center of the lung [17]. In our study, there was a positive association between the number of lesions and bilateral involvement of the lung with the duration of presenting complaint. In addition, the peripheral distribution of the lesions was associated with a short duration of presenting complaint (< 4 days) while diffuse pattern of distribution was associated with a relatively longer duration of presenting complaint (≥ 4 days). As regard the association between typical imaging lesions and duration of presenting complaint, no significant association could be detected between multifocal GGO, predominant consolidation, mixed pattern, or linear opacities and the duration of presenting complaint. The later results are non-concordant with the findings reported by Pan et al. [14]. They found that pulmonary consolidation is rare in the early stages of COVID-19 and increased with the progression of the disease. We cannot argue phases of disease progression as all CT scans included in our study were done relatively earlier in the disease course; however, we confirm that all typical findings can occur in the early stage. The crazy-paving pattern showed a positive association with increased duration of presenting complaint (p value = 0.002). All CT exams showing crazy-paving pattern were done after 5 days from the initial complaint. This finding is consistent with the reported findings in previous studies [15, 17–19, 25, 29].

Based on concurrent literature, older age, and co-existing comorbidities might be risk factors for the poor prognosis of COVID-19 patients [36]. In our study, we found an association between bilateral lesion distribution and diffuse lung involvement with the older age group (≥ 50 years old). Our cases showed more extensive lesions in the older age group (≥ 50 years old) than the younger age group (< 50 years old). This finding is matching the results reported by Chan et al. [37]. On the other hand, no significant association could be detected in our study between typical CT patterns and increased patient age. This contradicts what was reported by Li et al. that younger patients tended to have GGO while older patients tended to have more pulmonary consolidation. They admitted the presence of consolidation as a sign of a bad prognosis in elderly patients [17].

## Conclusion

The most common CT feature of COVID-19 was multifocal ground glass opacity. Atypical features like cavitation and pleural effusion can occur early in the course of the disease. Our cases showed more extensive lesions with bilateral and diffuse patterns of distribution in the older age group and with increased duration of presenting complaint. There was a significant association between crazy-paving pattern and increased duration of presenting complaint. No significant association could be detected between any CT pattern and increased patient age.

## Data Availability

The datasets used and/or analyzed during the current study are available on reasonable request.

## Abbreviations

COVID-19: Coronavirus disease 2019
rt RT-PCR: Real-time reverse-transcriptase polymerase chain reaction
CT: Computed tomography
GGO: Ground glass opacity
ACE2: Angiotensin-converting enzyme 2
DAD: Diffuse alveolar damage
ARDS: Acute respiratory distress syndrome
